# Privacy-Preserving Electrocardiogram Monitoring for Intelligent Arrhythmia Detection †

**DOI:** 10.3390/s17061360

**Published:** 2017-06-12

**Authors:** Junggab Son, Juyoung Park, Heekuck Oh, Md Zakirul Alam Bhuiyan, Junbeom Hur, Kyungtae Kang

**Affiliations:** 1 Department of Computer Science, Kennesaw State University, Marietta, GA 30060, USA; json@kennesaw.edu; 2 Sustainable Management Strategy, Korea Expressway Corporation, Gimcheon 39660, Korea; juyoung@ex.co.kr; 3 Department of Computer Science and Engineering, Hanyang University, Ansan 15588, Korea; hkoh@hanyang.ac.kr; 4 Department of Computer and Information Sciences, Fordham University, Bronx, NY 10458, USA; mbhuiyan3@fordham.edu; 5 Department of Computer Science and Engineering, Korea University, Seoul 02841, Korea

**Keywords:** body sensor networks, biomedical computing, electrocardiography, arrhythmia detection, communication system security, privacy of patients

## Abstract

Long-term electrocardiogram (ECG) monitoring, as a representative application of cyber-physical systems, facilitates the early detection of arrhythmia. A considerable number of previous studies has explored monitoring techniques and the automated analysis of sensing data. However, ensuring patient privacy or confidentiality has not been a primary concern in ECG monitoring. First, we propose an intelligent heart monitoring system, which involves a patient-worn ECG sensor (e.g., a smartphone) and a remote monitoring station, as well as a decision support server that interconnects these components. The decision support server analyzes the heart activity, using the Pan–Tompkins algorithm to detect heartbeats and a decision tree to classify them. Our system protects sensing data and user privacy, which is an essential attribute of dependability, by adopting signal scrambling and anonymous identity schemes. We also employ a public key cryptosystem to enable secure communication between the entities. Simulations using data from the MIT-BIH arrhythmia database demonstrate that our system achieves a 95.74% success rate in heartbeat detection and almost a 96.63% accuracy in heartbeat classification, while successfully preserving privacy and securing communications among the involved entities.

## 1. Introduction

Cyber physical systems have emerged as a promising paradigm for enriching the interactions between physical and cybernetic components. Recent advances in sensing technology and smart devices, which are the most important devices facilitating cyber-physical systems, have drastically altered the shape of the current healthcare environment, while presenting numerous opportunities and challenges in patient monitoring and assistance. This novel paradigm enables patients to monitor their physical conditions using smart devices [1], which has been particularly useful for chronic diseases that can become life threatening, such as high blood pressure, hypernatremia and various heart diseases that are common globally [2]. By monitoring a chronic disease, a patient can deal with this transformation at an early stage. In particular, monitoring arrhythmias is of high importance, because they are an extremely common initial symptom of cardiac arrest or myocardial infarction.

**Figure 1:**
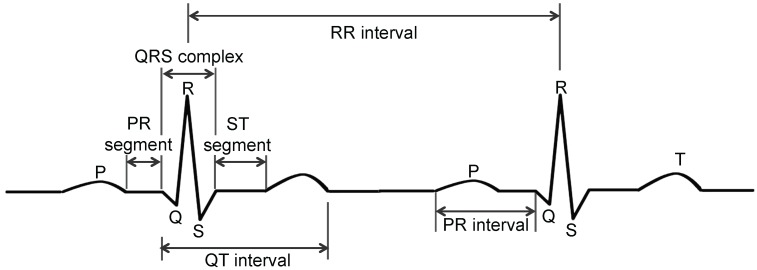
Structure of ECG.

Arrhythmia can be detected by analyzing an electrocardiogram (ECG), which measures the rate and regularity of heartbeats and is characterized by five peaks and valleys labeled P, Q, R, S and T (see Figure 1). The amplitude and duration of the P-Q-R-S-T wave provides information regarding the heart disease. The position and distance of the PR interval and segment, the ST interval and segment and the QT interval and QRS complex can be used in a diagnosis [3].

Several successful ECG monitoring systems have been developed [2,3,4,5,6]. These systems possess many advantages, such as allowing for ambulatory patient care, resulting in safer and more affordable healthcare. However, ECGs are primarily interpreted by medical experts, and most patients themselves cannot obtain the information firsthand. Thus, considerable attention has recently been devoted to the computerized automatic analysis of heart activity, which includes both the detection of the heartbeat in an electrocardiogram and the classification of its type. Recent advances in portable devices, such as smartphones and tablet PCs, also make it possible for users to more easily self-monitor their ECG status and classification results [4].

In order to exploit this potential, we propose a novel and intelligent ECG monitoring system (I-ECG) that analyzes and interprets heart activity by automatically detecting and classifying heartbeats based on the Pan–Tompkins [7] and C4.5 algorithms [8,9,10]. Although this was developed to be effective, simple to use, sustainable and reliable, it cannot be directly applied in real-world applications, owing to the following security and privacy problems. First, individuals close to the patients can illegally obtain the ECG signal during the communication between the sensors and the smartphone. Second, a decision support server (DSS) can manipulate collected ECG signals, as well as the status of a particular user. The health information that can be obtained through these security flaws is sensitive personal data, and thus, it could be abused for monetary purposes. In addition, the privacy of patients could be invaded by the abuse of this information.

It should be noted that many articles treat the privacy of patients as a primary concern in the usage of personal healthcare systems [11,12,13], and some of these assert that patient privacy should be preserved at all times by law [12]. Therefore, healthcare systems must be designed with consideration given to the security, in order to provide justifiably trusted services. Simultaneously, the system should be designed to safeguard the privacy of patients.

Contributions: The main objective of this study is to develop a privacy-preserving I-ECG that detects arrhythmia in the early stage. The contributions can be summarized as follows:

- We outline the guidelines for an intelligent ECG monitoring system and service model, and propose an algorithm for automatic detection and classification of arrhythmia, which realize intelligent monitoring as a decision support system.
- Additional computations performed by the body sensors to enable encryption of ECG data to protect it from attackers generate heat on the sensors, which can result in discomfort for the patient. To address this problem, we have developed an efficient and effective solution for encryption of ECG data in the body sensors. This solution selects the cipher with minimal overhead among the ciphers considered as candidates for our environment. In addition, our solution protects the patients’ privacy against attackers, including protection of data at the DSS, as well as monitoring stations by adopting changeable pseudonyms.
- We simulated our detection and classification algorithm for decision support using the MIT-BIH arrhythmia database [14], and the simulation results demonstrate that the DSS achieved an overall accuracy of 95.74%, with a sensitivity of 97.21% and a specificity of 94.26% for heartbeat detection. The DSS also achieved an overall accuracy of 96.63%, with a sensitivity of 95.44% and a specificity of 97.82% for heartbeat classification including privacy preservation measures.
- We demonstrate that our scheme exhibits a high efficiency compared with conventional cryptographic algorithms and an enhanced robustness against inside attackers who have access to the DSS, as well as against outside attackers who have the ability to eavesdrop on data from wireless communications.

The remainder of this paper is organized as follows. In Section 2, we briefly review related work regarding ECG monitoring systems and applications, with the focus on security and privacy. In Section 3, we describe the system models, threat models and security objectives. We describe our primary contribution, which is the privacy-preserving ECG monitoring system, in Section 4. In Section 5, we outline our system implementation. In Section 6, we evaluate and simulate our proposed system. Finally, we provide our conclusions in Section 7.

## 2. Related Work

Since the 1970s, researchers worldwide have been developing diagnostic systems that enable patients to make ECG recordings at home and transfer data to a cardiologist [2,3,4,5,6]. Research regarding remote ECG monitoring systems continues today, with smartphones being employed. Essentially, remote ECG monitoring systems [5,15,16] require only a simple architecture between the sensor device and a smartphone. The system extracts ECG signals through a lead cable, transmits the signal data via Bluetooth and then processes and displays the ECG waveform on a personal computer [15] or smartphone [5,16]. The system displays the ECG waveform on the device for remote monitoring. However, it does not transmit the ECG waveform to a medical expert. Therefore, the patient effectively receives no real-time medical service.

Systems on a healthcare server expand the fundamental remote monitoring system by offering an option to store and access ECG data. With the increasing popularity of Internet access through mobile phones, these systems provide an ideal platform between remote monitoring systems and patients. To display the ECG, many studies have employed a web component through a PC [17,18], single-chip microcomputer [19] or smartphone [2,18,20].

One proposed design for a remote monitoring “tele-medicine” system and web server consists of the client side, a general packet radio service (GPRS) modem and the server side [18]. The client side could be interpreted as the combination of the ECG, collection equipment, user interface and microprocessor. The GPRS modem, which is used to transmit the ECG signals, provides a large geographical mobility coverage range. The server side is divided into the back and front ends. The server front end is used to display the ECG signal on the web for patients and doctors, and the back end is designed to receive data from the GPRS modem and store it in a database. However, such designs are inadequate for medical experts, because they do not provide the information required to analyze the ECG signals.

In the recent past, many studies have applied QRS detection algorithms to healthcare servers. Such systems can record ECG signals on a web server and facilitate their analysis. One prototype uses a mobile phone as a gateway for transmitting measured ECG data back to the medical cloud using 3G mobile telecommunications or WiFi [6]. The system can also calculate the beats of the heart as RR intervals, which is the time between consecutive R-waves, on the mobile phone. Another system offers not only QRS detection, but also a priority-based alarm messaging service [4].

Although these approaches facilitate the convenient measurement and analysis of body status, they still encounter problems with security and privacy. As a number of healthcare applications deal with physical information, which constitutes sensitive personal information, data leakage and invasions of privacy in health monitoring systems are significant issues [11,12,13]. In 2012, Ma et al. proposed a simple, but effective security solution for ECG signals based on an ECG compression algorithm [21]. Regarding security and privacy challenges in mobile healthcare, Lu proposed an efficient user-centric privacy access scheme based on attribute-based access control and a novel privacy-preserving scalar product computation [22]. This scheme employs a body sensor node that can monitor various types of health information, and it has sufficient resources to apply a widely-employed symmetric encryption scheme such as AES. On the other hand, in this work, we focus on a body sensing device that only monitors an ECG, and thus, a smaller sensing device can be employed, which is more comfortable for patients. Because patients wear the sensing device for the entire day, it is an advantage to use a smaller device. Regarding this aspect, the scheme described above based on a symmetric key and hash functions requires more circuits and larger devices to handle the required cryptographic functions. Therefore, it remains a challenge to design a secure and privacy-preserving scheme for resource-constrained remote healthcare monitoring systems that utilize body sensors of a reduced size.

More recently, there have been attempts to deal with the use of ECG for identity recognition and biometric authentication [23,24]. For instance, Peter et al. [23] described the design and implementation steps required to realize an ECG-based authentication system in body area sensor networks and utilized ECG features for this purpose. Tan et al. [24] focused rather on enhancing the effectiveness and robustness of a biometric recognition system using a combination of random forest and wavelet distance measure classifiers. The ECG application investigated in this study is slightly different and has different design objectives. It is more oriented towards the secure transmission of ECG data to remote servers for analysis and to the application of machine learning to arrhythmia recognition. Enhancing the extent to which arrhythmia can be accurately detected is one of the eventual objectives of our study, and the energy is not our primary concern, because decisions are made on a remote server. Regarding networks, we are considerably interested in secure delivery over wide-area networks, rather than data transmission over body-area networks.

## 3. System Models, Threat Models and Security Objectives

This section describes the system models, threat models and security objectives of our system.

### 3.1. System Model

We are particularly interested in recording and accumulating ECG data for each patient over a long period of time, performing supervised learning based on the key ECG features and intelligent heartbeat classification for early and automatic detection of arrhythmia (we used a classifier based on a decision tree, which is constructed using the C4.5 learning algorithm). The crucial design consideration is the level of accuracy with which arrhythmia can be recognized. To achieve the desired accuracy, it is imperative that all of the acquired ECG data should be stored in the permanent storage of the DSS (i.e., database) and not on mobile devices with limited storage space.

**Figure 2:**
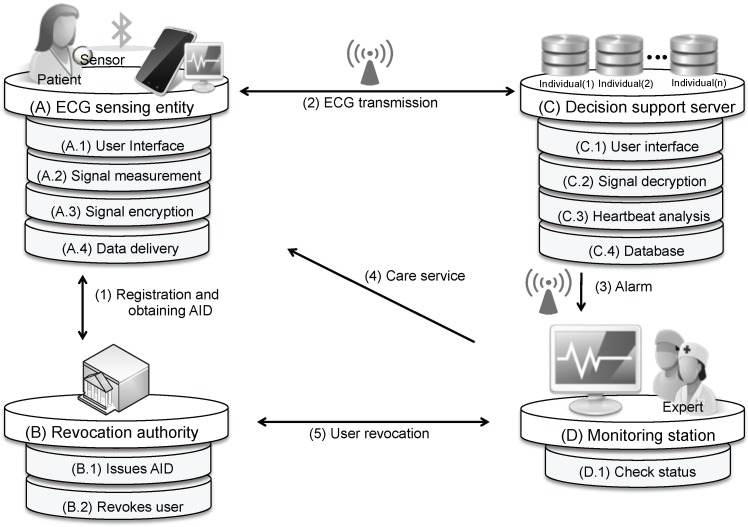
Overview of the proposed system architecture. AID, anonymous ID.

Our system comprises four components: a revocation authority, an ECG sensing entity, a decision support server (DSS) and a monitoring station, as depicted in Figure 2. The patient is able to lead a normal life while the sensor continuously acquires ECG data and sends them to a Bluetooth-enabled smartphone. The smartphone relays the data to the DSS, which associates incoming data with the records for the patient in a database and analyzes them. The heartbeat is detected using the Pan–Tompkins algorithm, which has been shown to be an effective QRS detection scheme [25], and then classified using a decision tree. If the system identifies congestive heart failure or an irregular heartbeat, then an alarm is sent over the Internet connection to a monitoring station, where medical personnel can carry out appropriate actions.

#### 3.1.1. Revocation Authority

We assume the existence of a revocation authority (RA) to hide a user’s identity, while being able to make it available during an emergency. The RA has three roles in the proposed scheme. First, it manages each user’s identity using a related anonymous ID (AID). Second, it reveals a user’s identity and contact information in an emergency, following a request by the monitoring station. Third, it issues certification for each entity’s public key. As the RA plays an important role in user identity management and public key confirmation, we treat the RA as a trusted third party. A government agency could set up and manage the RA for public welfare purposes.

#### 3.1.2. ECG Sensing Entity

**Figure 3:**
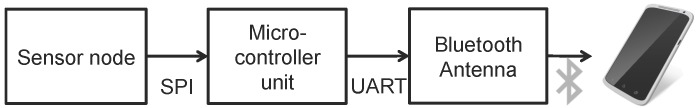
ECG signal measurement and delivery.

The smartphone of a patient collects ECG signals every day. Figure 3 illustrates the process of signal measurement and delivery. A sensor node is attached to the patient’s body. The signal from the sensor node is converted to a digital value using an analog-to-digital (ADC) converter and then sent to a microcontroller unit (MCU) through a serial peripheral interface, which is used primarily to communicate between chips. The signal at the MCU is sent to a Bluetooth module using a universal asynchronous receiver and transmitter. Subjects can view their ECG through a graphical interface designed for their mobile device. Although it is possible to perform self-monitoring, the ECG signal should also be transmitted to a DSS, because a typical layman cannot independently interpret the signal. Accordingly, the signal on the smart device is transmitted over a wireless network.

#### 3.1.3. Decision Support Server

The DSS analyzes multiple aspects of the ECG. As a convenience for medical experts, it acts as a monitoring station for decision support by detecting and classifying the heartbeat. Heartbeat detection in an ECG primarily depends on the QRS complex. However, QRS detection by itself is not sufficient for heartbeat detection, which must precede the recognition of features for detecting arrhythmia. One of the significant contributions of this study is the method of detecting the P-wave using the QRS complex, which is in turn detected by the Pan–Tompkins algorithm [7] (consisting of a band-pass filter, a differentiator and an integrator over a moving window). Eventually, the use of the P-wave with the QRS complex leads to the accurate detection of heartbeats related to arrhythmia.

#### 3.1.4. Monitoring Station

The monitoring station (MS) provides a graphical user interface. Therefore, both the patient and the expert operators can remotely check the signal. The patient, experts and a few others who have access rights can view the ECG, heart rate and patient profile through the web or a mobile device application. Moreover, experts using the MS can also view and determine the signal conditions of many patients through the web application. If an expert identifies a dangerous condition, then the MS immediately contacts medical services.

### 3.2. Threat Model

In the scope of this work, we consider normal users, including servers, as potential adversaries. Adversaries have limited capabilities, in that they can only access publicly-available information, including information from wireless communications. This is no more information than can be accessed by normal users of the ECG monitoring system. Thus, we consider that adversaries do not have the ability to distinguish the originator of data eavesdropped from a wireless communication. In the case of servers, we assume an honest-but-curious model that accurately follows a provided protocol, but may attempt to obtain information from the communication session or stored data. In addition, we do not consider that the adversaries can easily compromise other entities in order to obtain the identity of users or other valuable information. Based on these conditions, the following attacks can be carried out by adversaries.

- Eavesdropping: Essentially, we assume that an attacker has the ability of eavesdropping on data during transmissions. Wireless communications, including Bluetooth, which is used to transmit the sensing data, are vulnerable, because the transmitted data can be leaked by the major function hooking scheme of the windows kernel driver [26,27]. Thus, an attacker close to the sensing system can eavesdrop on the sensing data and the identity of a user via the weak point of our system.
- Leaking: We treat the DSS and service provider as potential attackers. The ECG analysis results and identity of an individual user could be leaked by the service providers or system managers of the DSS and MS, respectively. We only focus on cases where health information is leaked along with the identity of the relevant user.
- Tracking: After obtaining data (raw ECG data or an ECG analysis) by eavesdropping or leaking, an attacker could attempt to determine the relation between the data and the user. In addition, the attacker could attempt to trace a particular user by determining the relations between pseudonyms.

The collected sensing data for detecting arrhythmias is personal information for the user, and it could be used by an attacker in various ways. One possible scenario is selling the collected data for profit. Pharmaceutical companies or clinics could use such data for targeted marketing. In addition, the patient may not want his/her illness to be known to others. Such types of attacks are only possible if the attacker could obtain both the health information and the identity of the user.

### 3.3. Security Objectives

Depending on the system and threat models described earlier, we define two security objectives.

- Communication security: An attacker close to a user cannot obtain sensing signals from the wireless communication between the MCU and the smart device, nor between the smart device and the DSS.
- Privacy preservation: An attacker cannot establish a link between a particular user and their sensing signal or analysis results from the DSS. In the case of an emergency, the personal information of a user can be disclosed. During this step, an attacker can obtain only a limited amount of data from the server. In other words, from a collection of personal information provided and a pseudonym, the attacker cannot distinguish the ECG information of a particular user.

A simple method of preserving user privacy is to employ a pseudonym as an anonymous identity for communication, because this can easily hide the relation between data and a user’s identity. However, an attacker can easily obtain the identity of a certain user through long-term observation. Many previous schemes have mentioned the traceability of a single pseudonym [28,29,30,31]. In our system model, the attacker can obtain contact information for the pseudonym in an emergency. All of the patient’s information from before and after the emergency will be leaked if a single pseudonym-based approach is applied, and thus, the security model has to deal with forward and backward privacy. To deal with an emergency, a user’s identity should be revealed to receive medical service. Otherwise, an inside attacker can determine the relation between the user’s identity and the sensing signal. The forward privacy indicates that the attacker cannot determine a relation between sensing signals after an emergency. The backward privacy indicates that the attacker cannot determine a relation between sensing signals before the emergency.

## 4. System Architecture for Privacy-Preserving Intelligent ECG Monitoring

In this section, we propose an intelligent ECG monitoring system incorporating privacy preservation. The proposed scheme comprises two stages. First, we design a secure sensing signal encryption scheme using a conventional public key cryptosystem, to protect the sensing data transferred from the sensor to the MS via a smart device. Second, we present an AID scheme to preserve the user privacy during the arrhythmia recognition process. This also hides the user information from experts at the MS, but this can be revealed in an emergency. Before describing the proposed scheme, we present the notations used in this paper in Table 1.

### 4.1. Setup

Each user generates and uses a key and a pseudonym for each session to ensure privacy. One session is the duration of a pseudonym and the corresponding key. By changing these frequently, the system can provide stronger privacy protection. The length of each session is flexible. If a user wants a higher level of privacy, he/she can adjust to make the duration shorter. The user also can adjust to make the duration longer in the case that they require efficient operation.

When a user first establishes a connection between the MCU of a body sensor network and a smart device, the MCU and the smart device share a symmetric session key $\left\{k_{s}\right\}$ to deal with the limited storage of the MCU.

The RA, DSS, MS and the smart device of a user use a public key cryptosystem to establish secure communications. The public/private key pairs for the entities are $\left(pk_{RA},sk_{RA}\right),\left(pk_{DSS},sk_{DSS}\right),\left(pk_{MS},sk_{MS}\right)$ and $\left(pk_{U},sk_{U}\right)$, respectively.

### 4.2. Revocation Authority

Each user has a unique ID, and the user registers this along with his/her contact information to deal with emergencies. To generate an AID, the user encrypts their ID using a public key of the RA and sends this to the RA. At this point, we employ a set of session pseudonyms such that each user uses one pseudonym for only one session, to deal with forward and backward privacy in the single pseudonym approach [31].

The RA generates a set of unique pseudonyms $PS=\{p_{1},p_{2},\cdots ,p_{\ell }\}$, and computes the AID $A_{ID}={\left\{a_{j}\right\}}_{1\leq j\leq \ell }$ with its signature as:

$$a_{j}=E_{pk_{U}}\left\{H(ID|\right|p_{j})\},S_{sk_{RA}}\left\{a_{j}\right\},1\leq j\leq \ell$$

where *ℓ* is a natural number greater than one, which is defined by the user. To address the trade-off between privacy and efficiency, where the privacy is enhanced by changing the pseudonyms more frequently, we allow the user to define the frequency and *ℓ* while using the application. However, each session should be longer than 30 minutes to ensure an accurate analysis result.

The RA stores $\left(ID,A_{ID}\right)$ pairs, encrypts these using the user’s public key and then sends them to the user’s smart device. At this point, our system only uses the public key to encrypt the $\left(ID,A_{ID}\right)$ pairs. Therefore, an attacker cannot obtain the $A_{ID}$ used for an actual communication between the user and the DSS. Thus, using the user’s public key does not affect the privacy of the system. Finally, the user sends sensing signals to the DSS, preserving privacy by using one of the AIDs. As the user cannot receive services when an incorrect AID is used, a legal AID that is generated by the RA should be entered. After receiving the $A_{ID}$, the user generates a set of keys ${\left\{k_{i}\right\}}_{a\leq i\leq \ell }$.

In case of an emergency, the MS sends the AID to the RA. The RA then determines the patient information from the AID list and performs the step of connecting the patient with a doctor. Our scheme employs the RA as an additional entity to decentralize secret information and increase the effectiveness of privacy preservation.

### 4.3. ECG Sensing

To transfer the sensing signal securely, we use a stream cipher that consists of a pseudo-random generator (PRG) and a bitwise exclusive OR operator [32]. A sensor installed on the human body typically consists of a resource-constrained and battery-powered device. Moreover, considerable computations result in the production of more heat, which can be problematic in terms of patient safety. Furthermore, a body sensor requires a powerful processor and a large capacity battery to apply cryptography to its various functions, which would increase the size of the body sensing system and interfere with its day-to-day usage. Accordingly, we propose a simple and secure method that minimizes the encryption overhead and protects sensing data during transfer.

The MCU of the user generates the keystream $KS_{s}$ using the PRG such that $KS_{s}=PRG\left(k_{s}\right)$. Then, it computes the exclusive OR operation for the ECG signal $SS_{i}$ as $ES_{s}=KS_{s}⊕SS_{i}$, where $ES_{s}$ denotes the encrypted ECG signal. After receiving this, the smart device of the user performs the same operation as the MCU to decrypt the ECG signal, and the user can monitor their own ECG on selected smart devices.

To send the sensing signal safely, the user generates a key stream $KS_{i}$ using the PRG such that $KS_{i}=PRG\left(k_{i}\right)$ and encrypts $SS_{i}$ using $KS_{i}$ as $ES_{i}=KS_{i}⊕SS_{i}$. Subsequently, the user selects an $a_{i}$ randomly and sends the received signal stream to the DSS to detect arrhythmias using the ECG. The first time the user employs a choice of $a_{i}$, they send $a_{i},S_{sk_{RA}}\left\{a_{i}\right\}$ and $E_{pk_{DSS}}\left\{k_{i}\right\}$ for validity. Subsequently, the user sends the signal stream to the DSS as $a_{i}\left|\right|ES_{i}.$

For privacy reasons, we use a set of pseudonyms and a set of keys. If a user only uses a single pseudonym or key, then the DSS can easily trace that user. When a user’s identity is revealed in an emergency, the DSS can obtain all of the stored ECG signals and analysis results, as well as future information for the user. Therefore, users should periodically change their pseudonyms and keys to minimize leaked data after an emergency. After a received pseudonym is exhausted, the user requests a novel set of pseudonyms from the RA.

The DSS detects arrhythmias using continuous ECG streaming from users, and the pseudonyms transform that stream into a discontinuous signal. Thus, we should consider the effect of pseudonyms on the detection process. Consequently, we describe the simulation results of our scheme in Section 6, based on 30-minute of ECG signals, and show that we can ensure 96.63% accuracy if the pseudonym changing period is longer than half an hour.

### 4.4. Analysis of an ECG for Arrhythmia Detection

The DSS should first decrypt the signal stream to analyze the ECG. To achieve this, the DSS decrypts the key signal using its private key and obtains $k_{i}$. By obtaining $k_{i}$, the DSS can generate the same $KS_{i}$ as the MCU and decrypt the ECG signal as $SS_{i}=KS_{i}⊕ES_{i}$. Using $SS_{i}$, the DSS analyzes the multiple aspects of the ECG.

#### 4.4.1. Heartbeat Detection and Feature Extraction

**Figure 4:**
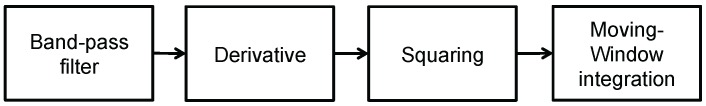
Process of the Pan–Tompkins algorithm.

**Figure 5:**
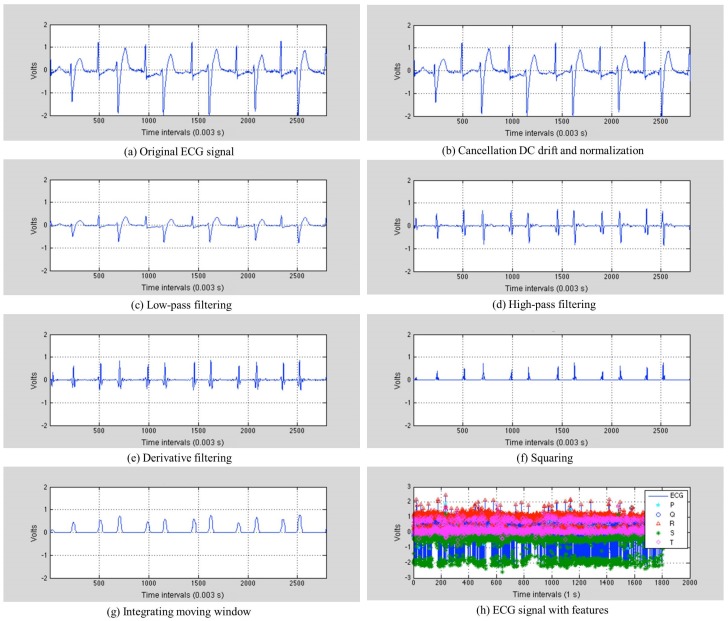
Results of feature extraction with step-by-step output of the Pan–Tompkins algorithm.

First, heartbeats are detected using the determined QRS complexes and P-waves. Figure 4 depicts the process of the Pan–Tompkins algorithm, and Figure 5 [25] illustrates the results of feature extraction, with the step-by-step output of the algorithm performed on Record 200 in the MIT-BIH arrhythmia database. Figure 5a shows the original ECG signal. The original signal is normalized by the mean value, as shown in Figure 5b. The band-pass filter is created by combining a low-pass filter with a high-pass filter. This reduces noise such as muscle noise, 60-Hz interference, baseline wander and T-wave interference in the ECG signal. The differential equation for the low-pass filter is:

(1) $$\begin{array}{cc} y(nT_{s})= & 2y\left(nT_{s}-T_{s}\right)-y\left(nT_{s}-2T_{s}\right)+x\left(nT_{s}\right)\\ & -x\left(nT_{s}-6T_{s}\right)+x\left(nT_{s}-12T_{s}\right), \end{array}$$

where $T_{s}$ denotes the sampling period, *x* is the amplitude of the *n*-th ECG sample and *y* is the amplitude after filtering. The difference equation for the high-pass filter is:

(2) $$\begin{array}{cc} y(nT_{s})= & 32x(nT_{s}-16T_{s})\\ & -[y\left(nT_{s}-T_{s}\right)+x\left(nT_{s}\right)-x\left(nT_{s}-32T_{s}\right)]. \end{array}$$

The DSS sets the low-pass filter with a cutoff frequency of 11 Hz and the high-pass filter with a cutoff frequency of 5 Hz, as shown in Figure 5c,d. After being filtered, the ECG signal is differentiated to provide slope information using the following differential equation:

(3) $$\begin{array}{cc} y(nT_{s})= & \frac{1}{8T_{s}}[-x\left(nT_{s}-2T_{s}\right)-2x\left(nT_{s}-T_{s}\right)\\ & +2x\left(nT_{s}+T_{s}\right)+x\left(nT_{s}+2T_{s}\right)], \end{array}$$

Equation (3) approximates the ideal derivative of frequencies up to 30 Hz, and Figure 5e presents the results of the derivative. This is then squared point by point, making all of the data points in the processed signal positive and emphasizing the higher frequencies, as shown in Figure 5f. The differential equation for this squaring is:

(4) $$\begin{array}{ccc} y(nT_{s}) & = & {\left[x\left(nT_{s}\right)\right]}^{2}. \end{array}$$

Integrating the moving window provides waveform feature information, which is added to the slope of the R-wave. This is achieved using the following differential equation:

(5) $$\begin{array}{cc} y(nT_{s})= & \frac{1}{N_{s}}[x\left(nT_{s}-\left(N_{s}-1\right)T_{s}\right)\\ & +x\left(nT_{s}-\left(N_{s}-2\right)T_{s}\right)+\cdots +x\left(nT_{s}\right)], \end{array}$$

where $N_{s}$ is the number of samples. This produces “re-echo mountaintops”, as depicted in Figure 5g, where each of the peaks corresponds to a heartbeat. The DSS finds the QRS complex in the heartbeat, where Q is the starting point, S is the ending point and R is the peak. The DSS then determines the P-wave using the QRS complex. This is observed between the S point of the current heartbeat and the Q point of the next heartbeat. The DSS divides this range into two, and uses the peak in the second subrange as the point P. Figure 5h depicts the sample results for the QRS complex and P-wave detection from Record 200 in the MIT-BIH arrhythmia database. The QRS complex and P-waves observed are all used for extracting the features from the heartbeats, and descriptions of these features are provided in Table 2.

The RR interval is used for assessing the ventricular rate. The DSS calculates the heart rate using the RR interval value. This is typically calculated using one of two algorithms, either the Fox and Haskell formula [33] or the mathematical formula described in [34]. The heart rate provides an easy cardiovascular measurement, particularly in comparison with invasive and noninvasive procedures used to estimate the stroke volume and cardiac output. To provide accurate health information, we display the maximum and resting heart rates. The maximum heart rate is the highest heart rate achieved during maximal exercise and is calculated as follows:

(6) $$\begin{array}{c} MHR=220-age. \end{array}$$

There are many methods for calculating the predicted maximum heart rate, including those of Tanaka et al. Fox and Haskell, Robergs and Landwehr, Gulati and Lund. We use Fox and Haskell’s formula, because this is widely adopted for the heart rate (max). The basal or resting heart rate is measured as follows:

(7) $$\begin{array}{c} BHR=60/RRinterval, \end{array}$$

when the patients are relaxed, but awake in a naturally temperate environment, and should have neither recently exerted themselves nor been subject to stress or a surprise. The DSS applies this basal heart rate formula.

#### 4.4.2. Learning Different Types of Heartbeats Using a C4.5 Algorithm

The DSS classifies the individual heartbeats in the ECG based on decision tree learning, which is one of the most widely-employed classification techniques. Its classification accuracy is competitive with other learning methods, and it is considerably efficient. The learned classification model is represented as a tree, called a decision tree. We trained the decision tree based on the C4.5 algorithm from Quinlan [8], which can provide prominent results, readability, flexibility and efficiency. The Iterative Dichotomiser 3 (ID3) [35] algorithm is mostly used for training the decision tree. This makes statistical-based decisions and is therefore less sensitive to errors in individual training examples. Although the ID3 algorithm is used in various domains, it is not applied to our server, because the discrete-valued target function domain cannot be applied to the ID3 algorithm. Therefore, our server uses the C4.5 algorithm, which is an extension of the basic ID3 algorithm. The pseudocode of this learning algorithm is presented in Algorithm 1.

**Algorithm 1** C4.5 algorithm for arrhythmia learning.1:**function** DecisionTree(*D*, *A*, *T*)     ▷ *D*: training dataset, *A*: feature set, *T*: decision tree2: **if** *D* contains only training examples of the same heartbeat type $c_{j}\in C$ **then**3:  Make *T* a leaf node labeled with heartbeat type $c_{j}$;      ▷ *C*: heartbeat types4: **else if** A=Ø **then**5:  Make *T* a leaf node labeled with heartbeat type $c_{j}$, which is the most frequent heartbeat type in *D*; 6: **else**         ▷ *D* contains examples belonging to a mixture of heartbeat types7:   $p_{i}$=impurityEval-1(D); ▷ We select a single feature to partition *D* into subsets so that each subset purer8:   **for** Each feature $A_{i}\in A\left(=\left\{A_{1},A_{2},\ldots ,A_{k}\right\}\right)$ **do**9:    $p_{i}$=impurityEval-$2(A_{i},D)$;10:  **end for**11:  Select $A_{g}\in \left\{A_{1},A_{2},\ldots ,A_{k}\right\}$ that provides the biggest impurity reduction, computed using $p_{0}-p_{i}$; 12:  **if** $(p_{0}-p_{g})<\mathrm{threshold}$ **then**     ▷ $A_{g}$ does not significantly reduce impurity $p_{0}$
13:   Make *T* a leaf node labeled with $c_{j}$, the most frequent heartbeat type in *D*;14:  **else**                    ▷ $A_{g}$ is able to reduce impurity $p_{0}$
15:   Make *T* a decision node on $A_{g}$; 16:   Let the possible values of $A_{g}$ be $v_{1},v_{2},\ldots ,v_{m}$. Partition *D* into *m* disjoint subsets $D_{1},D_{2},\ldots ,D_{m}$ based on the *m* values of $A_{g}$;17:   **for** Each $D_{j}\in D\left(=\left\{D_{1},D_{2},\ldots ,D_{m}\right\}\right)$ **do**18:    **if** $D_{j}\neq Ø$ **then**
19:     Create a branch (edge) node $T_{j}$ for $v_{j}$ as a child node of *T*; 20:     DecisionTree$\left(D_{j},A-\left\{A_{g}\right\},T_{j}\right)$;21:    **end if**22:   **end for**23:  **end if**24: **end if**25:**end function**


In particular, a decision tree *T* simply partitions the training dataset *D* into disjoint subsets, so that each subset is as pure as possible (of the same heartbeat type), by considering a feature set *A*. The learning of a tree is typically performed using the divide-and-conquer strategy, which recursively partitions the data to produce the tree. Initially, all of the examples are at the root. As the tree grows, the examples are subdivided recursively. In this study, we assume that every feature in *D* takes discrete values.

The stopping criteria of the recursion are presented in Lines 1–4 in Algorithm 1. The algorithm stops when all of the training examples in the current data are of the same heartbeat type, or when every feature is used along the current tree path. In tree learning, each successive recursion selects the best feature to partition the data at the current node according to the values of the feature. The best feature is selected based on a function that aims to minimize the impurity after the partitioning (Lines 7–11). In other words, it maximizes the purity. The key in decision tree learning is thus the choice of the impurity function, which is employed in Lines 7, 9 and 11 in Algorithm 1. The recursive recall of the algorithm is given in Line 20, which takes the subset of the training examples at the node for further partitioning to extend the tree. This is a greedy algorithm, with no backtracking. The process of node creation is irreversible; no modification is possible once a node is created.

The most popular impurity functions used for decision tree learning are information gain and information gain ratio, which are used in C4.5 as two portions. Let us first discuss information gain, which can be extended slightly to produce information gain ratio. The information gain measure is based on the entropy function from information theory:

(8) $$\begin{array}{c} Entropy\left(D\right)=-\sum_{j=1}^{\left|C\right|}\mathrm{Pr}(c_{j})\log_{2}\mathrm{Pr}\left(c_{j}\right),\mathrm{where}\left(\sum_{\left|C\right|}^{j=1}\mathrm{Pr}\left(c_{j}\right)=1\right). \end{array}$$

$C=\{c_{1},c_{2},\ldots ,c_{\left|C\right|}\}$ is a set of |C| heartbeat types and $\mathrm{Pr}(c_{j})$ is the probability of heartbeat type $c_{j}$ to occur in the dataset *D*. This is given by the number of examples of heartbeat type $c_{j}$ in *D* divided by the total number of examples in *D*.

#### 4.4.3. Heartbeat Classification for Arrhythmia Detection Using a Decision Tree

The DSS classifies the individual heartbeats in the ECG using a decision tree learned by the C4.5 algorithm, as mentioned above, and then sends the AID and analysis results to the MS. Each of the ECG records from the MIT-BIH arrhythmia database can contain a maximum of 11 heartbeat types, consisting of a normal beat (N) and 10 abnormal beats: left bundle branch block beat (L), right bundle branch block beat (R), atrial premature beat (A), aberrated atrial premature beat (a), premature ventricular contraction beat (V), fusion of ventricular and normal beat (F), ventricular flutter wave beat (!), atrial escape beat (e), ventricular escape beat (E) and paced beat (P). Table 3 summarizes the 10 different types of ventricular and atrial heartbeat that we are targeting to classify for arrhythmia detection, which are all annotated for signal quality and rhythm changes.

## 5. Implementation

### 5.1. Mobile System

We designed an actual system for the simulation. As our scheme includes a real-time streaming signal with a feature extraction and classification process performed by a server, it is important to prove that our scheme can operate in real applications. Therefore, our implementation focused on two concerns. First, as mentioned earlier, we implemented our scheme to show that it is applicable to the remote monitoring setting. Second, our implementation shows that it can detect and classify heartbeats with a high accuracy.

**Figure 6:**
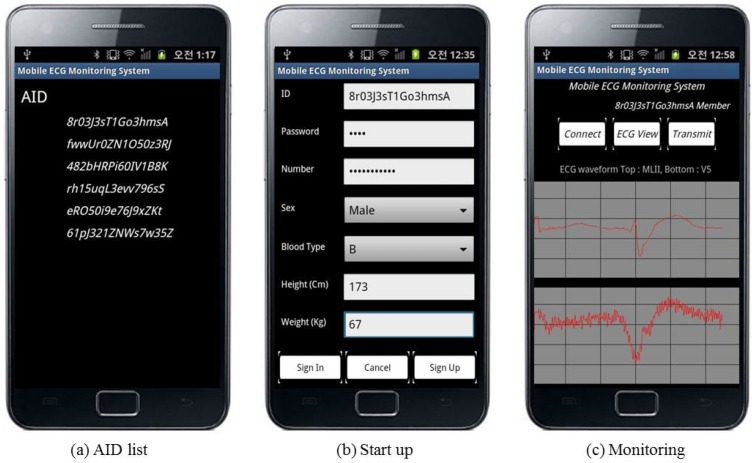
Results of mobile device implementation.

The system software consisted of a mobile system and a DSS. The mobile system was implemented in Java, using the Android SDK 2.3.3 (Google, Mountain View, CA, USA). We selected the Android platform because it is open source. Moreover, it has a large developer community that writes applications for it and provides code portability. We selected Apache 2.0 (Apache Software Foundation, Wakefield, MA, USA) to host the web application, which links to MySQL. Figure 6 illustrates the implementation of the mobile system.

### 5.2. DSS Specification

**Figure 7:**
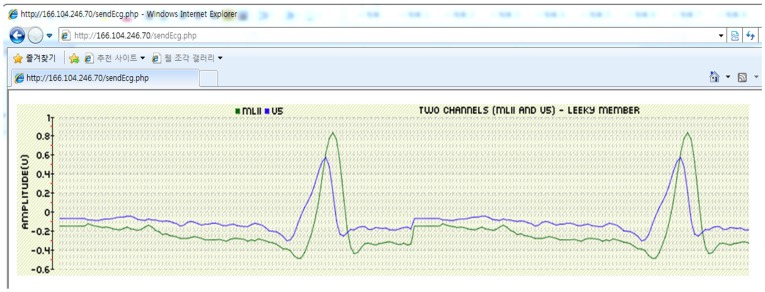
Results of web application implementation.

The DSS connects with an individual database and analyzes the heartbeat based on the QRS complexity algorithm. It displays graphical ECG waveforms through a web application. Figure 7 depicts the results from the web application. We employed several frameworks for the implementation of the DSS, including PHP 5.1.4 (Zend Technologies Ltd. Louisville, CO, USA), pChart 2.1.3 and MySQL 5.0.51a (Oracle Corporation, Santa Clara, CA, USA). We used PHP as the primary framework, owing to its operational speed and cross-platform compatibility. As a PHP class, pChart is an oriented framework designed for creating aliased charts. We used a MySQL database to maintain records for the patients and experts affiliated with the health monitoring program.

## 6. Evaluation and Results

In this section, we evaluate our scheme based on the three criteria of security, efficiency and accuracy.

### 6.1. Security

Concerning the security and privacy of the ECG monitoring system, we adopted a PRG-based stream cipher to protect the ECG signal, and we conceal the identity of the user from the DSS using a pseudonym system. To verify that our system is secure, we verify that our scheme satisfies the security objectives.

We define the indistinguishability of encryptions from the perspective of communication security, which is the first security objective.

**Definition** **1**  (Indistinguishability of encryptions [36])**.** *An encryption scheme,* (G,E,D)*, which consists of a key generator G, encryption E and decryption D, exhibits indistinguishable encryptions. For every polynomial-size circuit family* $\left\{C_{n}\right\}$*, every positive polynomial p, all sufficiently large n and every* $x,y\in {\left\{0,1\right\}}^{poly(n)}$ *(i.e.,* |x|=|y|),

$$|Pr\left[C_{n}\left(E_{G(1^{n})}\left(x\right)\right)=1\right]-Pr\left[C_{n}\left(E_{G(1^{n})}\left(y\right)\right)=1\right]|<\frac{1}{p(n)}.$$

*The probability in these terms is taken over the internal coin tosses of the algorithms G and E.*

A symmetric key encryption is semantically secure if and only if it exhibits indistinguishable encryption [36]. Thus, we prove that our encryption scheme has indistinguishability.

**Claim** **1.**  *Suppose that there exist PRGs that exhibit robustness against polynomial-size circuits, and our scheme adopts one of these. Then, the encryption scheme based on the PRG satisfies Definition 1.*

**Proof.**  First, we formally explain our encryption scheme. The key for the security parameter *n* is a uniformly-distributed *n* bit-long string, denoted by $k_{i}$. To encrypt a sensing stream $SS_{i}$, the encryption algorithm uses the key $k_{i}$ as a seed for a PRG, denoted by *g*, which stretches seeds of length *n* into sequences of length $|SS_{i}|$. The ciphertext is obtained by a bit-by-bit exclusive OR of $SS_{i}$ and $g(k_{i})$. Decryption is performed in the same manner.

We show that this encryption scheme satisfies Definition 1. Intuitively, this follows from the hypothesis that *g* is a PRG and the fact that $SS_{i}⊕U_{|SS_{i}|}$ is uniformly distributed over ${\left\{0,1\right\}}^{|SS_{i}|}$. In particular, in order to obtain a contradiction, suppose that for some polynomial-size circuit family $\left\{C_{n}\right\}$, a polynomial *p* and infinitely many *n*’s, it holds that:

$$\begin{array}{ccc} |Pr\left[C_{n}\left(SS_{i}⊕g\left(U_{n}\right)\right)=1\right]-Pr\left[C_{n}\left(SS_{j}⊕g\left(U_{n}\right)\right)=1\right]| & > & \frac{1}{p(n)}, \end{array}$$

where $U_{n}$ is uniformly distributed over ${\left\{0,1\right\}}^{n}$ and $|SS_{i}|=|SS_{j}|=m=poly\left(n\right)$. On the other hand,

$$Pr\left[C_{n}\left(SS_{i}⊕g\left(U_{n}\right)\right)=1\right]=Pr\left[C_{n}\left(SS_{j}⊕g\left(U_{n}\right)\right)=1\right].$$

Thus, without loss of generality:

$$\begin{array}{ccc} |Pr\left[C_{n}\left(SS_{i}⊕g\left(U_{n}\right)\right)=1\right]-Pr\left[C_{n}\left(SS_{i}⊕U_{m}\right)\right)=1]| & > & \frac{1}{2\cdot p(n)}. \end{array}$$

By incorporating $SS_{i}$ into the circuit $C_{n}$, we obtain a circuit that distinguishes $U_{m}$ from $g(U_{n})$, in contradiction of our hypothesis regarding the pseudorandomness of *g*.  ☐

Therefore, the stream cipher derived from the PRG is semantically secure [36]. The only problem regarding the security of our system is the key. If the key stored in the MCU and smart device is leaked, then a third party could obtain the ECG signal and the relationship between the signal and the user. To store the key securely, tamper-resistant memory can be attached to the MCU. In addition, key leakage resulting from weaknesses in the smart device system, such as malware infections, constitutes a security issue for the device itself. The treatment of this is beyond the scope of this study.

Next, we verify that our scheme can preserve user privacy. This privacy preservation is the second security objective.

**Definition** **2**  (Polynomial-time indistinguishability of pseudonym [37])**.** *Suppose that there exist PRGs that exhibit robustness against polynomial-size circuits. Then, a pseudonym generated using such a PRG exhibits polynomial-time indistinguishability.*

*Two pseudonyms* $X\overset{def}{=}{\left\{X_{n}\right\}}_{n\in N}$ *and* $Y\overset{def}{=}{\left\{Y_{n}\right\}}_{n\in N}$ *that are uniformly distributed over* ${\left\{0,1\right\}}^{n}$ *by the PRG are indistinguishable in polynomial time if for every probabilistic polynomial-time algorithm D, every positive polynomial* p() *and all sufficiently large n’s, it holds that:*

$$\begin{array}{c} |Pr\left[D\left(X_{n},1^{n}\right)=1\right]-Pr\left[D\left(Y_{n},1^{n}\right)=1\right]|<\frac{1}{p(n)}. \end{array}$$

Our system only provides the information required to detect arrhythmia, such as age. The AIDs used for privacy preservation are generated by the PRG, and thus, the AIDs exhibit polynomial-time indistinguishability according to Definition 2. Therefore, our scheme is secure against tracking attacks, as well as data leakage resulting from the DSS. However, the DSS and MS will be able to obtain a user’s identity from the RA when an instance of arrhythmia is detected. In this case, they can obtain the real identity of a user and their ECG signals that are stored in the server. In terms of privacy, changing pseudonyms is useful for minimizing the data leakage, because only the ECG data stored under the current pseudonym will be leaked in an emergency. If an expert requires further information regarding the user, the expert can request additional pseudonyms from the RA. In addition, another security issue is the manipulation of ciphertexts. An attacker could manipulate the service by transferring ciphertexts in order to disrupt a smooth use of services. This problem can occur not only under the XORing circumstance, but also under other encryption circumstances, as this is a problem of the networks. Therefore, we do not consider this problem in this paper. Such integrity issues could easily be addressed by periodically applying a message authentication code (MAC).

### 6.2. Efficiency

To evaluate the efficiency of our scheme, we simulated it against other well-known encryption schemes. We implemented our experiment on an Intel (R) core (TM) 2 Quad processor, running at 2.66 GHz, 4.00 GB of RAM and an HDD Serial ATA 3.0 Gbit/s drive with an 8 MB buffer. All algorithms are implemented using Python 2.7.9 on Linux Ubuntu 12.04 LTS 32 bit (Canonical Ltd., London, UK), and we used the PyCrypto library version 2.6.1 for the ciphers (AES, DES and RC4). We used a 128-bit key with cipher feedback (CFB) mode for AES and a 64-bit key with CFB mode for DES. For the PRG, we used “os.urandom()”, which is the basic library of Python that is suitable for cryptographic usage. We measured the encryption and decryption time for 300 s of an ECG signal. The volume of this ECG signal is approximately 4 Mbyte. However, this could vary in accordance with the attached sensors. All experimental results represent the average over 10 trials.

Table 4 shows the comparison of the encryption and decryption times between ciphers. The results show that our scheme is considerably efficient compared with most of the other cryptography schemes, although RC4, which consists of a key scheduling algorithm and bit-wise exclusive OR, exhibits a similar efficiency to our scheme. However, RC4 is known to be insecure, owing to a weakness of the key scheduling algorithm [38]. Therefore, it is better to use a cryptographic PRG as a key expanding algorithm. In this case, a developer can select a PRG algorithm based on the condition of an MCU.

In addition, we only used RSA with a 1024-bit key to encrypt the set of pseudonyms for mobile devices, not for body sensors. This requires 1.416 ms of computational overhead for encryption and 17.97 ms for decryption. As the pseudonym communication is rarely used, we can say that this overhead is reasonable for privacy preservation.

### 6.3. Accuracy of Arrhythmia Classification

#### 6.3.1. ECG data

To evaluate our classifier in terms of accuracy, we used the MIT-BIH arrhythmia database [14], which contains 48 30-minute excerpts of two-channel ambulatory ECG recordings, obtained from 47 patients studied at the BIH Arrhythmia Laboratory between 1975 and 1979. Twenty-three recordings were selected at random from a set of 4000 24-hour ambulatory ECG recordings, collected from a mixed population of inpatients (approximately 60%) and outpatients (approximately 40%) at Boston’s Beth Israel Hospital. The remaining 25 recordings were selected from the same set, to include less common, but clinically-significant arrhythmias (such as complex ventricular, junctional and supraventricular arrhythmias and conduction abnormalities) that would not be represented well in a small random sample.

The recordings were digitized at 360 samples per second per channel, with 11 bit resolution, over a 10-mV range. Two or more cardiologists independently annotated each record, and disagreements were resolved to obtain computer-readable reference annotations for each beat (approximately 110,000 annotations in all) included in the database. We considered nine records from the database, numbered as 106, 107, 109, 115, 122, 207, 212, 221 and 233. Table 5 provides a statistical overview of these heartbeat types for the nine records. The column headed @ contains totals for the following events: beat not classified during learning (?), change in signal quality (*), isolated QRS-like artifact (|) and rhythm change (+).

#### 6.3.2. Evaluation

We used the three standard metrics of sensitivity (*Se.*), specificity (*Sp.*) and accuracy (*Acc.*) to quantify the server performance. Sensitivity refers to the ability of a test to correctly identify the results of a classified heartbeat with a positive result and is defined as follows:

(9) $$\begin{array}{c} Se.=N_{TP}/\left(N_{TP}+N_{FN}\right), \end{array}$$

where $N_{TP}$ represents the number of true positives and $N_{FN}$ represents the number of false negatives. Specificity refers to the ability of a test to correctly identify the results of a classified type without a positive result and is given by:

(10) $$\begin{array}{c} Sp.=N_{TN}/\left(N_{TN}+N_{FP}\right), \end{array}$$

where $N_{TN}$ represents the number of true negatives and $N_{FP}$ represents the number of false positives. Accuracy refers to the ability of a test to correctly identify the results of a classified type both with and without positive results. It reflects both the sensitivity and specificity and is expressed as:

(11) $$\begin{array}{c} Acc.=\left(N_{TP}+N_{TN}\right)/\left(N_{TP}+N_{TN}+N_{FN}+N_{FP}\right). \end{array}$$

We evaluated the performance of the server in two aspects for the analysis of the heartbeat. The first aspect of the evaluation related to heartbeat detection, which affects the classification performance. The 11 types of heartbeat listed in Table 3 were considered for evaluation, and Table 6 illustrates the performance for each of the nine records. For the nine records we considered from the MIT-BIH arrhythmia database, the server achieved an overall accuracy of 95.74%, with a sensitivity of 97.21% and a specificity of 94.26%. It is noted that Record 107 exhibits many abnormal beats, with an unusually large, typically peaked P-wave, which resulted in a particularly low rate of specificity of 50.05%. This result could be enhanced by a deeper analysis of the P-wave. However, this is beyond the scope of the present study.

The second aspect of the evaluation concerned heartbeat classification for arrhythmia detection. We used a 10-fold cross-validation method to test the classification [39]. The classification performance is summarized in Table 6. The server achieved an overall accuracy of 96.63%, with a sensitivity of 95.44% and a specificity of 97.81%. If we consider an ECG recorded for a longer period of time, the analysis for the diagnosis could be more accurate.

## 7. Conclusions

In this paper, we have proposed a privacy-preserving intelligent ECG monitoring system for early arrhythmia detection and described its implementation. The major steps required for accurate recognition of arrhythmia are (1) the accurate detection of heartbeats and (2) defining the significant features of those heartbeats and extracting them to recognize various types of heartbeats, which involves leveraging the effectiveness of a machine learning algorithm and employing it for decision making. The steps we invoked to achieve the required accuracy are as follows. First, we proposed and applied a scheme for P-wave detection, along with the well-known Pan–Tompkins algorithm, to enhance the accuracy of heartbeat detection at the first step. Second, this enhanced accuracy in the heartbeat detection naturally has a positive influence on the subsequent feature extraction process. Third, we introduced a classifier based on a decision tree and trained it using a C4.5 algorithm, which resulted in several enhancements to the former ID3 algorithm, by considering various combinations of ECG features. We rigorously investigated the effects of these combinations on the accuracy of the decision making using a decision tree and determined one of the feature sets featuring the best compromises by using a brute force search in the third step. As a result, we achieved an average accuracy of 96.63% in recognizing arrhythmia for nine example records from the MIT-BIH arrhythmia database that we considered for our experiments, which is superior to the results (95.8%) achieved in our preliminary studies.

In addition, we designed a simple and effective sensing data scrambling scheme to ensure the security of streamed sensing data and adopted AIDs to preserve user privacy. Using our proposed system, medical experts can capture the intermittent ECG waveforms that may reveal or lead to a more serious problem. An accurate diagnosis of arrhythmia based on a heartbeat detection and classification algorithm can be achieved in real time, which is beneficial for remote medical care. This system represents a low-cost solution, which could be affordable across medical environments.

It is clear that no scheme of this sort is fully proven until it has been tried in practice. However, the implementation and testing of medical systems is especially fraught with difficulties, from patient confidentiality to legal concerns, and cannot reasonably be undertaken until there is as much confidence as possible in the techniques and associated parameters being deployed. It is this confidence that we aim to build in this paper, through simulations based on offline data. 

## Figures and Tables

**Table 1:** Notations.

Notation	Description
*k*	A symmetric session key
(pk,sk)	Public, private key pair
$p_{\ell }$	A pseudonym
PS	A set of pseudonyms, $PS=\{p_{1},p_{2},\cdots ,p_{\ell }\}$
$a_{\ell }$	An anonymous ID
$A_{ID}$	A set of anonymous IDs, $A_{ID}={\left\{a_{j}\right\}}_{1\leq j\leq \ell }$
H(·)	A cryptographic hash function
PRG(·)	A pseudo-random number generator
$E_{k}\left\{\right\}$	An encryption function using key *k*
$S_{k}\left\{\right\}$	A signature function using key *k*
KS	A key stream
ES	An encrypted ECG signal using key *k*

**Table 2:** Description of the features used for heartbeat classification.

Feature	Description
P-position	Position of P point
Q-position	Position of Q point
R-position	Position of R point
S-position	Position of S point
P-value	Value of P point (amplitude)
Q-value	Value of Q point (amplitude)
R-value	Value of R point (amplitude)
S-value	Value of S point (amplitude)
PR-distance	Distance between P point and R point
RR-interval	Distance between consecutive R-waves
*MHR*	Maximum heart rate
*BHR*	Resting heart rate

**Table 3:** Description of heartbeat types.

Arrhythmia		The Rest	
Type	Description	Type	Description
L	Left bundle branch block beat	N	Normal beat
R	Right bundle branch block beat	?	Beat not classified during learning
A	Atrial premature beat	*	Change in signal quality
a	Aberrated atrial premature beat	|	Isolated QRS-like artifact
V	Premature ventricular contraction beat	+	Rhythm change
F	Fusion of ventricular and normal beat		
!	Ventricular flutter wave beat		
e	Atrial escape beat		
E	Ventricular escape beat		
P	Paced beat		

**Table 4:** Simulation results (ms).

	AES	DES	RC4	Ours
Encryption	1220.54	1366.30	47.09	46.80
Decryption	1222.32	1362.98	48.86	45.64

**Table 5:** Characteristics of each record selected from the MIT-BIH arrhythmia database.

Record Number	N	L	R	A	a	V	F	!	e	E	P	@
106	1507	-	-	-	-	520	-	-	-	-	-	71
107	-	-	-	-	-	59	-	-	-	-	2078	3
109	-	2492	-	-	-	38	2	-	-	-	-	3
115	1953	-	-	-	-	-	-	-	-	-	-	9
122	2476	-	-	-	-	-	-	-	-	-	-	3
207	-	1457	86	107	-	105	-	472	-	105	-	53
212	923	-	1825	-	-	-	-	-	-	-	-	15
221	2031	-	-	-	-	396	-	-	-	-	-	35
223	2029	-	-	72	1	473	14	-	16	-	-	38

**Table 6:** Results of heartbeat detection and classification for arrhythmia recognition (sensitivity (*Se.*), specificity (*Sp.*) and accuracy (*Acc.*)).

		Heartbeat Detection			Arrhythmia Detection	
Rec.	*Se.* (%)	*Sp.* (%)	*Acc.* (%)	*Se.* (%)	*Sp.* (%)	*Acc.* (%)
106	93.99	99.80	96.90	95.80	98.00	96.90
107	99.86	50.05	74.96	93.00	98.30	95.65
109	99.64	99.84	99.74	93.30	94.40	93.85
115	99.59	100.00	99.80	97.80	100.00	98.90
122	99.96	100.00	99.98	99.90	100.00	99.95
207	86.83	99.90	93.37	92.60	98.80	95.70
212	99.53	99.85	99.69	94.20	97.80	96.00
221	98.21	99.67	98.94	94.20	95.70	94.95
233	97.30	99.22	98.26	98.20	97.30	97.75
**Average**	**97.21**	**94.26**	**95.74**	**95.44**	**97.81**	**96.63**
